# Pointwise approximation by a Durrmeyer variant of Bernstein-Stancu operators

**DOI:** 10.1186/s13660-016-1291-x

**Published:** 2017-01-27

**Authors:** Lvxiu Dong, Dansheng Yu

**Affiliations:** Department of Mathematics, Hangzhou Normal University, Hangzhou, Zhejiang 310036 China

**Keywords:** 41A25, 41A35, Bernstein-Stancu type operators, pointwise and global estimates, inverse results

## Abstract

In the present paper, we introduce a kind of Durrmeyer variant of Bernstein-Stancu operators, and we obtain the direct and converse results of approximation by the operators.

## Introduction

For any $f\in C_{ [ 0,1 ] }$, the corresponding Bernstein operators and Bernsetin-Durrmeyer operators are defined by

1.1 $$ B_{n} ( f,x ) :=\sum_{k=0}^{n}f \biggl( \frac{k}{n} \biggr) p_{nk} ( x ) $$

and

1.2 $$ D_{n} ( f,x ) := ( n+1 ) \sum_{k=0}^{n}p_{nk} ( x ) \int_{0}^{1}f ( t ) p_{nk} ( t ) \,dt, $$

respectively, where $p_{nk} ( x ) :=\binom{n}{k}x^{k} ( 1-x ) ^{n-k}$, $k=0,1,\ldots,n$. Both $B_{n} ( f,x ) $ and $D_{n} ( f,x ) $ have played very important roles in approximation theory and computer science. There are many generalizations of the operators $B_{n} ( f,x ) $ and $D_{n} ( f,x ) $. Among them, Gadjiev and Ghorbanalizadeh [1] introduced the following new generalized Bernstein-Stancu type operators with shifted knots:

1.3 $$ S_{n,\alpha,\beta} ( f,x ) := \biggl( \frac{n+\beta _{2}}{n} \biggr) ^{n}\sum _{k=0}^{n}f \biggl( \frac{k+\alpha_{1}}{n+\beta_{1}} \biggr) q_{nk} ( x ) , $$

where $x\in A_{n}:= [ \frac{\alpha_{2}}{n+\beta_{2}},\frac {n+\alpha _{2}}{n+\beta_{2}} ] $, and

$$ q_{nk} ( x ) :=\binom{n}{k} \biggl( x-\frac{\alpha_{2}}{n+\beta _{2}} \biggr) ^{k} \biggl( \frac{n+\alpha_{2}}{n+\beta_{2}}-x \biggr) ^{n-k},\quad k=0,1,\ldots,n, $$

with $\alpha_{k}$, $\beta_{k}$, $k=1,2$ positive numbers satisfying $0\leq \alpha_{1}\leq\beta_{1}$, $0\leq\alpha_{2}\leq\beta_{2}$. Obviously, when $\alpha_{1}=\alpha_{2}=\beta_{1}=\beta_{2}=0$, $S_{n,\alpha,\beta } ( f,x ) $ reduces to the classical Bernstein operators in (1.1), when $\alpha_{2}=\beta_{2}=0$, it reduces to the so-called Bernstein-Stancu operators which were introduced by Stancu [2]:

1.4 $$ B_{n,\alpha,\beta} ( f,x ) :=\sum_{k=0}^{n}f \biggl( \frac {k+\alpha }{n+\beta} \biggr) p_{nk} ( x ) . $$

Some approximation properties and generalizations of the operators $S_{n,\alpha,\beta}(f,x)$ can be found in [3–5].

Motivated by (1.3), we introduce the following generalization of the operators (1.2):

$$ \widetilde{S}_{n,\alpha,\beta} ( f,x ) := \biggl( \frac {n+\beta_{2}}{n} \biggr) ^{n}\sum_{k=0}^{n} \lambda_{nk}^{-1}q_{nk} ( x ) \int_{A_{n}}q_{nk} ( t ) f \biggl( \frac{nt+\alpha_{1}}{n+\beta _{1}} \biggr) \,dt, $$

where

$$ \lambda_{nk}= \int_{A_{n}}q_{nk} ( t ) \,dt,\quad k=0,1,\ldots,n, $$

and $\alpha_{k}$, $\beta_{k}$, $k=1,2$ positive numbers satisfying $0\leq \alpha_{1}\leq\beta_{1}$, $0\leq\alpha_{2}\leq\beta_{2}$.

By Lemma 1 in Section 2, we observe that $\widetilde{S}_{n,\alpha,\beta } ( f,x ) $ can be rewritten as follows:

$$ \widetilde{S}_{n,\alpha,\beta} ( f,x ) = \biggl( \frac{n+\beta _{2}}{n} \biggr) ^{2n+1}\sum_{k=0}^{n}q_{nk} ( x ) ( n+1 ) \int_{A_{n}}q_{nk} ( t ) f \biggl( \frac{nt+\alpha_{1}}{n+\beta _{1}} \biggr) \,dt. $$

Especially, when $\alpha_{1}=\alpha_{2}=\beta_{1}=\beta _{2}=0$, $\widetilde{S}_{n,\alpha,\beta} ( f,x ) $ reduces to the classical Bernstein-Durrmeyer operators in (1.2). Many authors have studied some special cases of the operators $\widetilde{S}_{n,\alpha,\beta} ( f,x ) $. For example, the case $\alpha_{1}=\alpha_{2}=\beta_{1}=0$ in [6] by Jung, Deo, and Dhamija, the case $\alpha_{1}=\beta_{1}=0 $ in [7] by Acar, Aral, and Gupta.

The main purpose of the present paper is to establish pointwise direct and converse approximation theorems of approximation by $\widetilde {S}_{n,\alpha ,\beta} ( f,x ) $. To state our result, we need some notations:

1.5 $$\begin{aligned}& \omega_{\varphi^{\lambda}}^{2} ( f,t ) =\sup_{0< h\leq t}\sup _{x\pm h\varphi^{\lambda}\in A_{n}}\bigl\vert \Delta_{h\varphi ^{\lambda}}^{2}f(x) \bigr\vert , \end{aligned}$$

1.6 $$\begin{aligned}& D_{\lambda}^{2}= \bigl\{ f\in C ( A_{n} ) ,f^{\prime}\in \mathit{A.C.}_{\mathrm{loc}},\bigl\Vert \varphi^{2\lambda}f^{\prime\prime}\bigr\Vert < +\infty \bigr\} , \\& K_{\varphi^{\lambda}} \bigl( f,t^{2} \bigr) =\inf_{g\in D_{\lambda }^{2}} \bigl\{ \Vert f-g\Vert +t^{2}\bigl\Vert \varphi ^{2\lambda }g^{\prime\prime}\bigr\Vert \bigr\} , \end{aligned}$$

1.7 $$\begin{aligned}& \overline{D}_{\lambda}^{2}= \bigl\{ f\in D_{\lambda}^{2}, \bigl\Vert f^{\prime\prime}\bigr\Vert < +\infty \bigr\} , \\& \overline{K}_{\varphi^{\lambda}} \bigl( f,t^{2} \bigr) =\inf _{g\in \overline{D}_{\lambda}^{2}} \bigl\{ \Vert f-g\Vert +t^{2}\bigl\Vert \varphi^{2\lambda}g^{\prime\prime}\bigr\Vert +t^{4/(2-\lambda)}\bigl\Vert g^{\prime\prime}\bigr\Vert \bigr\} , \end{aligned}$$

and $\varphi(x)=\sqrt{ ( x-\frac{\alpha_{2}}{n+\beta_{2}} ) ( \frac{n+\alpha_{2}}{n+\beta_{2}}-x ) }$, $0\leq\lambda \leq1$. It is well known (see [8], Theorem 3.1.2) that

1.8 $$ \omega_{\varphi^{\lambda}}^{2} ( f,t ) \sim K_{\varphi ^{\lambda }} \bigl( f,t^{2} \bigr) \sim\overline{K}_{\varphi^{\lambda}} \bigl( f,t^{2} \bigr) , $$

where $x\sim y$ means that there exists a positive constant *c* such that $c^{-1}y\leq x\leq cy$.

Our first result can be read as follows.

### Theorem 1

*Let* *f* *be a continuous function on* $A_{n}$, $\lambda\in [ 0,1 ] $ *be a fixed positive number*. *Then there exists a positive constant* *C* *only depending on* *λ*, $\alpha_{1}$, $\alpha_{2}$, $\beta_{1}$, *and* $\beta_{2}$ *such that*

1.9 $$ \bigl\vert \widetilde{S}_{n,\alpha,\beta} ( f,x ) -f ( x ) \bigr\vert \leq C \biggl( \omega_{\varphi^{\lambda}} \biggl( f, \frac{\delta_{n}^{1-\lambda} ( x ) }{\sqrt{n}} \biggr) +\omega \biggl( f,\frac{1}{n} \biggr) \biggr) , $$

*where* $\delta_{n}(x)=\varphi(x)+1/\sqrt{n}\sim \max \{ \varphi (x),1/\sqrt{n} \} $, *and* $\omega ( f,t ) $ *is the usual modulus of continuity of* *f* *on* $A_{n}$.

Throughout the paper, *C* denotes either a positive absolute constant or a positive constant that may depend on some parameters but not on *f*, *x*, and *n*. Their values may be different at different locations.

For the converse result, we have the following.

### Theorem 2

*Let* *f* *be a continuous function on* $A_{n}$, $0<\alpha<\frac {2}{2-\lambda}$, $0\leq\lambda\leq1$. *Then*

1.10 $$ \bigl\vert \widetilde{S}_{n,\alpha,\beta} ( f,x ) -f(x)\bigr\vert =O \bigl( \bigl( n^{-1/2}\delta_{n}^{1-\lambda }(x) \bigr) ^{\alpha} \bigr) $$

*implies that*

1.11 $$ (\mathrm{i})\quad \omega_{\varphi^{\lambda}}^{2} ( f,t ) =O \bigl(t^{\alpha }\bigr);\qquad (\mathrm{ii})\quad \omega ( f,t ) =O \bigl( t^{\alpha ( 1-\lambda/2 ) } \bigr) . $$

## Auxiliary lemmas

### Lemma 1

*We have*

2.1 $$ \lambda_{kn}= \int_{A_{n}}q_{nk} ( t ) \,dt= \biggl( \frac {n}{n+\beta _{2}} \biggr) ^{n+1}\frac{1}{n+1},\quad k=0,1,\ldots,n. $$

### Proof

For $p,q=1,2,\ldots$ , set

$$\begin{aligned} B^{\ast} ( p,q ) :=& \int_{A_{n}} \biggl( x-\frac{\alpha_{2}}{n+\beta_{2}} \biggr) ^{p-1} \biggl( \frac{n+\alpha_{2}}{n+\beta_{2}}-x \biggr) ^{q-1}\,dx \\ = & \int_{0}^{\frac{n}{n+\beta_{2}}}x^{p-1} \biggl( \frac{n}{n+\beta_{2}} -x \biggr) ^{q-1}\,dx. \end{aligned}$$

Then

$$\begin{aligned} B^{\ast} ( p,q ) =&\frac{q-1}{p} \int_{0}^{\frac{n}{n+\beta _{2}}}x^{p} \biggl( \frac{n}{n+\beta_{2}}-x \biggr) ^{q-2}\,dx \\ =&\frac{q-1}{p} \int_{0}^{\frac{n}{n+\beta_{2}}} \biggl( \frac {n}{n+\beta_{2}}x^{p-1}-x^{p-1} \biggl( \frac{n}{n+\beta_{2}}-x \biggr) \biggr) \biggl( \frac{n}{n+\beta_{2}}-x \biggr) ^{q-2}\,dx \\ =&\frac{q-1}{p}\cdot\frac{n}{n+\beta_{2}}B^{\ast} ( p,q-1 ) -\frac{q-1}{p}B^{\ast} ( p,q ) , \end{aligned}$$

which implies that

$$ B^{\ast} ( p,q ) =\frac{q-1}{p+q-1}\cdot\frac{n}{n+\beta _{2}}B^{\ast} ( p,q-1 ) . $$

Therefore,

$$\begin{aligned} \lambda_{kn} =&\binom{n}{k}B^{\ast} ( k+1,n-k+1 ) \\ =& \biggl( \frac{n}{n+\beta_{2}} \biggr) ^{n-k}\binom{n}{k} \frac{ ( n-k ) ( n-k-1 ) \cdots2\cdot1}{ ( n+1 ) n\cdots ( k+2 ) }B^{\ast} ( k+1,1 ) \\ =& \biggl( \frac{n}{n+\beta_{2}} \biggr) ^{n-k}\frac{k+1}{ ( n+1 ) }\int_{0}^{\frac{n}{n+\beta_{2}}}x^{k}\,dx \\ =& \biggl( \frac{n}{n+\beta_{2}} \biggr) ^{n+1}\frac{1}{n+1}. \end{aligned}$$

 □

### Lemma 2

*For any* $x\in A_{n}$, *we have*

2.2 $$ \widetilde{S}_{n,\alpha,\beta} \bigl( ( t-x ) ^{2},x \bigr) \leq \frac{C}{n}\delta_{n}^{2} ( x ) . $$

### Proof

Write

$$ \widetilde{D}_{n,\alpha,\beta} ( f,x ) := \biggl( \frac {n+\beta_{2}}{n} \biggr) ^{2n+1}\sum_{k=0}^{n}q_{nk} ( x ) ( n+1 ) \int_{A_{n}}q_{nk} ( t ) f ( t ) \,dt. $$

Then [7]

2.3 $$\begin{aligned}& \widetilde{D}_{n,\alpha,\beta} ( 1,x ) =1, \widetilde {D}_{n,\alpha,\beta} ( t,x ) =\frac{n}{n+2}x+\frac{n+2\alpha _{2}}{ ( n+2 ) ( n+\beta_{2} ) }, \\& \widetilde{D}_{n,\alpha,\beta} \bigl( t^{2},x \bigr) = \biggl( x- \frac{ \alpha_{2}}{n+\beta_{2}} \biggr) ^{2}\frac{n ( n-1 ) }{ ( n+2 ) ( n+3 ) } \\& \hphantom{\widetilde{D}_{n,\alpha,\beta} \bigl( t^{2},x \bigr) ={}}{}+\frac{n}{n+\beta_{2}} \biggl( x-\frac{\alpha_{2}}{n+\beta_{2}} \biggr) \frac{4n}{ ( n+2 ) ( n+3 ) } \\& \hphantom{\widetilde{D}_{n,\alpha,\beta} \bigl( t^{2},x \bigr) ={}}{}+ \biggl( \frac{n}{n+\beta_{2}} \biggr) ^{2} \frac{2}{ ( n+2 ) ( n+3 ) }+\frac{2n\alpha_{2}}{ ( n+2 ) ( n+\beta _{2} ) } \biggl( x-\frac{\alpha_{2}}{n+\beta_{2}} \biggr) \\& \hphantom{\widetilde{D}_{n,\alpha,\beta} \bigl( t^{2},x \bigr) ={}}{}+\frac{2n\alpha_{2}}{ ( n+2 ) ( n+\beta_{2} ) ^{2}}+ \biggl( \frac{\alpha_{2}}{n+\beta_{2}} \biggr) ^{2}, \end{aligned}$$

and

$$ \widetilde{D}_{n,\alpha,\beta} \bigl( ( t-x ) ^{2},x \bigr) \leq \frac{C}{n}\delta_{n}^{2} ( x ) . $$

By the facts that

2.4 $$\begin{aligned}& \widetilde{S}_{n,\alpha,\beta} ( 1,x ) =\widetilde {D}_{n,\alpha ,\beta} ( 1,x ) =1, \\& \widetilde{S}_{n,\alpha,\beta} ( t,x ) =\frac{n}{n+\beta _{1}}\widetilde{D}_{n,\alpha,\beta} ( t,x ) +\frac{\alpha_{1}}{n+\beta_{1}}, \end{aligned}$$

and

$$ \widetilde{S}_{n,\alpha,\beta} \bigl( t^{2},x \bigr) =\frac {n^{2}}{ ( n+\beta_{1} ) ^{2}} \widetilde{D}_{n,\alpha,\beta} \bigl( t^{2},x \bigr) + \frac{2n\alpha_{1}}{ ( n+\beta_{1} ) ^{2}}\widetilde{D}_{n,\alpha,\beta} ( t,x ) + \frac{\alpha _{1}^{2}}{ ( n+\beta_{1} ) ^{2}}, $$

we get

$$\begin{aligned} \widetilde{S}_{n,\alpha,\beta} \bigl( ( t-x ) ^{2},x \bigr) =&\frac{n^{2}}{ ( n+\beta_{1} ) ^{2}}\widetilde{D}_{n,\alpha ,\beta } \bigl( ( t-x ) ^{2},x \bigr) \\ &{}+ \biggl( \frac{2n^{2}x}{ ( n+\beta_{1} ) ^{2}}+\frac {2n\alpha_{1}}{ ( n+\beta_{1} ) ^{2}}-\frac{2nx}{n+\beta_{1}} \biggr) \widetilde{D}_{n,\alpha,\beta} ( t,x ) \\ &{}+\frac{\alpha_{1}^{2}}{ ( n+\beta_{1} ) ^{2}}-\frac{2\alpha _{1}x}{n+\beta_{1}}+x^{2}-\frac{n^{2}}{ ( n+\beta_{1} ) ^{2}}x^{2} \\ =&\frac{n^{2}}{ ( n+\beta_{1} ) ^{2}}\widetilde{D}_{n,\alpha ,\beta} \bigl( ( t-x ) ^{2},x \bigr) +\frac{ ( \beta _{1}^{2}+4\beta_{1} ) n+2\beta_{1}^{2}}{ ( n+\beta_{1} ) ^{2} ( n+2 ) }x^{2} \\ &{}+\frac{2\alpha_{1} ( \beta_{1}+\beta_{2}+2 ) n^{2}+2n\alpha _{1} ( \beta_{1}\beta_{2}+2\beta_{1}+2\beta_{2} ) +4\alpha _{1}\beta_{1}\beta_{2}}{ ( n+\beta_{1} ) ^{2} ( n+2 ) ( n+\beta_{2} ) }x \\ &{}+\frac{\alpha_{1}^{2}}{ ( n+\beta_{1} ) ^{2}} \\ \leq&\widetilde{D}_{n,\alpha,\beta} \bigl( ( t-x ) ^{2},x \bigr) + \frac{C}{n^{2}} \\ \leq&\frac{C}{n}\delta_{n}^{2} ( x ) . \end{aligned}$$

 □

### Lemma 3

*For any given* $\gamma\geq0$, *we have*

2.5 $$ \sum_{k=0}^{n}\biggl\vert \frac{k+\alpha_{2}}{n+\beta_{2}}-x\biggr\vert ^{\gamma}\bigl\vert q_{nk} ( x ) \bigr\vert \leq C\frac {\delta _{n}^{\gamma} ( x ) }{n^{\gamma/2}}, \quad x\in [ 0,1 ] . $$

### Proof

It was showed in [3] that

2.6 $$ \sum_{k=0}^{n}\biggl\vert \frac{k+\alpha_{1}}{n+\beta_{1}}-x\biggr\vert ^{\gamma}\bigl\vert q_{nk} ( x ) \bigr\vert \leq C\frac{ ( \delta_{n}^{\ast} ( x ) ) ^{\gamma}}{n^{\gamma /2}}, \quad x\in [ 0,1 ] , $$

where $\delta_{n}^{\ast} ( x ) :=\psi ( x ) +\frac {1}{\sqrt{n}}$ and $\psi ( x ) =\sqrt{x ( 1-x ) }$. We verify that

2.7 $$ \delta_{n}^{\ast} ( x ) \sim\delta_{n} ( x ) ,\quad x\in [ 0,1 ] . $$

In fact, when $x\in [ \frac{2\alpha_{2}+1}{n+\beta_{2}},\frac{ n-\beta_{2}+2\alpha_{2}}{n+\beta_{2}} ] $, we have

$$\begin{aligned}& \frac{1}{2}x\leq x-\frac{\alpha_{2}}{n+\beta_{2}}\leq x, \\& \frac{1}{2} ( 1-x ) \leq\frac{n+\alpha_{2}}{n+\beta _{2}}-x\leq 1-x. \end{aligned}$$

Thus,

$$ \psi ( x ) \sim\varphi ( x ) , $$

which implies (2.7) for $x\in [ \frac{2\alpha_{2}+1}{n+\beta _{2}},\frac{n-\beta_{2}+2\alpha_{2}}{n+\beta_{2}} ] $. When $x\in [ 0,\frac{2\alpha_{2}+1}{n+\beta_{2}} ) \cup ( \frac{n-\beta _{2}+2\alpha_{2}}{n+\beta_{2}},1 ] $, we have

2.8 $$ \delta_{n}^{\ast} ( x ) \sim\delta_{n} ( x ) \sim \frac{1}{\sqrt{n}}, $$

and thus (2.7) also holds.

Now, by (2.6) and (2.7), we have

$$\begin{aligned} \sum_{k=0}^{n}\biggl\vert \frac{k+\alpha_{2}}{n+\beta_{2}}-x\biggr\vert ^{\gamma}\bigl\vert q_{nk} ( x ) \bigr\vert \leq &\sum_{k=0}^{n}\biggl\vert \frac{k+\alpha_{2}}{n+\beta_{2}}-\frac {k+\alpha _{1}}{n+\beta_{1}}\biggr\vert ^{\gamma}\bigl\vert q_{nk} ( x ) \bigr\vert +\sum_{k=0}^{n} \biggl\vert \frac{k+\alpha_{1}}{n+\beta_{1}} -x\biggr\vert ^{\gamma}\bigl\vert q_{nk} ( x ) \bigr\vert \\ \leq&\frac{C}{n^{\gamma}}\sum_{k=0}^{n}\bigl\vert q_{nk} ( x ) \bigr\vert +C\frac{\delta_{n}^{\gamma} ( x ) }{n^{\gamma /2}} \\ \leq&C\frac{\delta_{n}^{\gamma} ( x ) }{n^{\gamma/2}}. \end{aligned}$$

 □

### Lemma 4

*For any* $x\in A_{n}$, *we have*

2.9 $$ \sum_{k=0}^{n}q_{nk} ( x ) ( n+1 ) \int_{A_{n}}\delta _{n}^{2} ( t ) q_{nk} ( t ) \,dt\leq C\delta _{n}^{2} ( x ) $$

*and*

2.10 $$ \sum_{k=0}^{n-1}q_{n-1,k} ( x ) n \int_{A_{n}}\delta _{n}^{-2} ( t ) q_{n+1,k+1} ( t ) \,dt\leq C\delta_{n}^{-2} ( x ) . $$

### Proof

By a similar calculation to that of Lemma 1, we have

2.11 $$ \int_{A_{n}}\varphi^{2} ( t ) q_{nk} ( t ) \,dt= \biggl( \frac{n}{n+\beta_{2}} \biggr) ^{n+3}\frac{ ( n-k+1 ) ( k+1 ) }{ ( n+3 ) ( n+2 ) ( n+1 ) }. $$

On the other hand, we have

$$\begin{aligned} \sum_{k=0}^{n} \biggl( \frac{k}{n}- \frac{k^{2}}{n^{2}} \biggr) q_{nk} ( x ) =& \biggl( \frac{n}{n+\beta_{2}} \biggr) ^{n-1} \biggl( x-\frac{\alpha_{2}}{n+\beta_{2}} \biggr) - \biggl( \frac{n}{n+\beta_{2}} \biggr) ^{n-1}\frac{ ( x-\frac{\alpha_{2}}{n+\beta_{2}} ) }{n} \\ &{}-\frac{n-1}{n} \biggl( \frac{n}{n+\beta_{2}} \biggr) ^{n-2} \biggl( x-\frac{\alpha_{2}}{n+\beta_{2}} \biggr) ^{2} \\ =&\frac{n-1}{n} \biggl( \frac{n}{n+\beta_{2}} \biggr) ^{n-2}\varphi ^{2} ( x ) . \end{aligned}$$

Therefore,

$$\begin{aligned} \sum_{k=0}^{n}q_{nk} ( x ) ( n+1 ) \int_{A_{n}}\delta _{n}^{2} ( t ) q_{nk} ( t ) \,dt \leq& 2\sum_{k=0}^{n}q_{nk} ( x ) ( n+1 ) \int_{A_{n}} \biggl( \varphi^{2} ( t ) + \frac{1}{n} \biggr) q_{nk} ( t ) \,dt \\ \leq&2\sum_{k=0}^{n}q_{nk} ( x ) \biggl( \frac{n}{n+\beta _{2}} \biggr) ^{n+3}\frac{ ( n-k+1 ) ( k+1 ) }{ ( n+3 ) ( n+2 ) } \\ &{}+ \frac{C}{n}\sum_{k=0}^{n}q_{nk} ( x ) \\ \leq&C\sum_{k=0}^{n}q_{nk} ( x ) \biggl( \frac{ ( n-k ) k}{n^{2}}+\frac{1}{n} \biggr) +\frac{C}{n} \\ \leq&C\delta_{n}^{2} ( x ) , \end{aligned}$$

which proves (2.9).

By Lemma 1, we have

$$\begin{aligned} n \int_{A_{n}}\delta_{n}^{-2} ( t ) q_{n+1,k+1} ( t ) \,dt \leq&Cn \int_{A_{n}} \bigl( \varphi^{-2} ( t ) +n \bigr) q_{n+1,k+1} ( t ) \,dt \\ \leq&Cn \biggl( \int_{A_{n}}\varphi^{-2} ( t ) q_{n+1,k+1} ( t ) \,dt+1 \biggr) \\ =&Cn \biggl( \frac{ ( n+1 ) n}{ ( k+1 ) ( n-k ) }\int_{A_{n}}q_{n-1,k} ( t ) \,dt+1 \biggr) \\ \leq&Cn \biggl( \frac{ ( n+1 ) }{ ( k+1 ) ( n-k ) }+1 \biggr) \\ \leq&Cn. \end{aligned}$$

Then

$$\begin{aligned} \sum_{k=0}^{n-1}q_{n-1,k} ( x ) n \int_{A_{n}}\delta _{n}^{-2} ( t ) q_{n+1,k+1} ( t ) \,dt \leq &Cn\sum_{k=0}^{n}q_{n-1,k} ( x ) \\ =&Cn\leq C\delta_{n}^{-2} ( x ) . \end{aligned}$$

Hence, (2.10) is proved. □

### Lemma 5

*If* *f* *is* *r* *times differentiable on* $[ 0,1 ] $, *then*

2.12 $$\begin{aligned} \widetilde{S}_{n,\alpha,\beta}^{(r)} ( f,x ) =& \biggl( \frac{ n+\beta_{2}}{n} \biggr) ^{2n+1} \biggl( \frac{n}{n+\beta_{1}} \biggr) ^{r} \frac{ ( n+1 ) !n!}{ ( n-r ) ! ( n+r ) !}\sum_{k=0}^{n-r}q_{n-r,k}(x) \\ &{}\times \int_{\frac{\alpha_{2}}{n+\beta_{2}}}^{\frac{n+\alpha_{2}}{n+\beta_{2}}}q_{n+r,k+r}(t)f^{(r)} \biggl( \frac{nt+\alpha_{1}}{n+\beta _{1}} \biggr) \,dt. \end{aligned}$$

### Proof

By using Leibniz’s theorem, we have

$$\begin{aligned} \widetilde{S}_{n,\alpha,\beta}^{(r)} ( f,x ) =& \biggl( \frac{ n+\beta_{2}}{n} \biggr) ^{2n+1}\sum_{i=0}^{r} \sum_{k=i}^{n-r+i}\binom {r}{i}\frac{(-1)^{r-i} ( n+1 ) !}{ ( k-i ) ! ( n-k-r+i ) !} \\ &{}\times \biggl( x-\frac{\alpha_{2}}{n+\beta_{2}} \biggr) ^{k-i} \biggl( \frac{n+\alpha_{2}}{n+\beta_{2}}-x \biggr) ^{n-k-r+i} \int_{\frac{\alpha _{2}}{n+\beta_{2}}}^{\frac{n+\alpha_{2}}{n+\beta_{2}}}q_{nk}(t)f \biggl( \frac{nt+\alpha_{1}}{n+\beta_{1}} \biggr) \,dt \\ =& \biggl( \frac{n+\beta_{2}}{n} \biggr) ^{2n+1}\sum _{k=i}^{n-r+i}\sum_{i=0}^{r} \binom{r}{i}\frac{(-1)^{r-i} ( n+1 ) !}{ ( n-r ) !}q_{n-r,k-i}(x) \\ &{}\times \int_{\frac{\alpha_{2}}{n+\beta_{2}}}^{\frac{n+\alpha_{2}}{n+\beta_{2}}}q_{nk}(t)f \biggl( \frac{nt+\alpha_{1}}{n+\beta_{1}} \biggr) \,dt \\ =& \biggl( \frac{n+\beta_{2}}{n} \biggr) ^{2n+1}\frac{ ( n+1 ) !}{ ( n-r ) !}\sum _{k=0}^{n-r}(-1)^{r}q_{n-r,k}(x) \\ &{}\times \int_{\frac{\alpha_{2}}{n+\beta_{2}}}^{\frac{n+\alpha_{2}}{n+\beta_{2}}}\sum_{i=0}^{r} \binom{r}{i}(-1)^{i}q_{n,k+i}(t)f \biggl( \frac{nt+\alpha_{1}}{n+\beta_{1}} \biggr) \,dt. \end{aligned}$$

Since

$$ \frac{d^{r}}{dt^{r}}q_{n+r,k+r}(t)=\sum_{i=0}^{r} \binom {r}{i}(-1)^{i}\frac{ ( n+r ) !}{n!}q_{n,k+i}(t), $$

we have

$$\begin{aligned} \widetilde{S}_{n,\alpha,\beta}^{(r)} ( f,x ) =& \biggl( \frac{n+\beta_{2}}{n} \biggr) ^{2n+1}\frac{ ( n+1 ) !n!}{ ( n-r ) ! ( n+r ) !}\sum_{k=0}^{n-r}q_{n-r,k}(x) \\ &{}\times\int_{\frac{\alpha_{2}}{n+\beta_{2}}}^{\frac{n+\alpha_{2}}{n+\beta_{2}}}(-1)^{r}q_{n+r,k+r}^{(r)}(t)f \biggl( \frac{nt+\alpha_{1}}{n+\beta_{1}} \biggr) \,dt. \end{aligned}$$

We obtain the required result by integrating by parts *r* times. □

Set

$$\begin{aligned}& \Vert f\Vert _{0}=\sup_{x\in A_{n}} \bigl\{ \bigl\vert \delta _{n}^{\alpha ( \lambda-1 ) }(x)f ( x ) \bigr\vert \bigr\} ; \\& C_{\alpha,\lambda}= \bigl\{ f\in C ( A_{n} ) ,\Vert f\Vert _{0}< +\infty \bigr\} ; \\& \Vert f\Vert _{1}=\sup_{x\in A_{n}} \bigl\{ \bigl\vert \delta _{n}^{ ( \frac{2}{2-\lambda}-\alpha ) ( 1-\lambda ) }(x)f^{\prime} ( x ) \bigr\vert \bigr\} ; \\& C_{\alpha,\lambda}^{1}= \bigl\{ f\in C_{\alpha,\lambda}, \Vert f\Vert _{1}< +\infty \bigr\} ; \\& \Vert f\Vert _{2}=\sup_{x\in A_{n}} \bigl\{ \bigl\vert \delta _{n}^{2+\alpha ( \lambda-1 ) }(x)f^{\prime\prime} ( x ) \bigr\vert \bigr\} ; \\& C_{\alpha,\lambda}^{2}= \bigl\{ f\in C_{\alpha,\lambda},f^{\prime } \in \mathit{A.C.}_{\mathrm{loc}},\Vert f\Vert _{2}< +\infty \bigr\} ; \\& K_{\alpha,\lambda}^{1} ( f,t ) =\inf_{g\in C_{\alpha,\lambda }^{1}} \bigl\{ \Vert f-g\Vert _{0}+t\Vert g\Vert _{1} \bigr\} ; \\& K_{\alpha,\lambda}^{2} ( f,t ) =\inf_{g\in C_{\alpha,\lambda }^{2}} \bigl\{ \Vert f-g\Vert _{0}+t\Vert g\Vert _{2} \bigr\} . \end{aligned}$$

### Lemma 6

*If* $0\leq\lambda\leq1$, $0<\alpha<2$, *then*

2.13 $$\begin{aligned}& \bigl\Vert \widetilde{S}_{n,\alpha,\beta} ( f ) \bigr\Vert _{1}\leq Cn^{1/ ( 2-\lambda ) }\Vert f\Vert _{0},\quad f\in C_{\alpha,\lambda}, \end{aligned}$$

2.14 $$\begin{aligned}& \bigl\Vert \widetilde{S}_{n,\alpha,\beta} ( f ) \bigr\Vert _{1}\leq C\Vert f\Vert _{1},\quad f\in C_{\alpha,\lambda }^{1}. \end{aligned}$$

### Proof

Firstly, we prove (2.13) by considering the following two cases.

*Case* 1. $x\in B_{n}:= [ \frac{\alpha_{2}+1}{n+\beta_{2}}, \frac{n+\alpha_{2}-1}{n+\beta_{2}} ] $. In this case, we have

$$ \varphi ( x ) \geq\min \biggl( \varphi \biggl( \frac{\alpha _{2}+1}{n+\beta_{2}} \biggr) , \varphi \biggl( \frac{n+\alpha_{2}-1}{n+\beta _{2}} \biggr) \biggr) \geq \frac{C}{\sqrt{n}}, $$

which means that

2.15 $$ \delta_{n}(x)\sim\varphi ( x ) \quad \text{for }x\in B_{n}. $$

By simple calculations, we have

2.16 $$ q_{nk}^{\prime} ( x ) =n\varphi^{-2} ( x ) \biggl( \frac{k+\alpha_{2}}{n+\beta_{2}}-x \biggr) q_{nk} ( x ) $$

and

2.17 $$\begin{aligned} \delta_{n} \biggl( \frac{nt+\alpha_{1}}{n+\beta_{1}} \biggr) =&\sqrt{ \biggl( t-\frac{\alpha_{2}}{n+\beta_{2}}+\frac{\alpha_{1}-\beta _{1}t}{n+\beta_{1}} \biggr) \biggl( \frac{n+\alpha_{2}}{n+\beta_{2}}-t+\frac {\beta _{1}t-\alpha_{1}}{n+\beta_{1}} \biggr) }+\frac{1}{\sqrt{n}} \\ =&\sqrt{\varphi^{2} ( t ) +O \biggl( \frac{1}{n} \biggr) }+ \frac {1}{\sqrt{n}}\sim\varphi ( t ) +\frac{1}{\sqrt{n}}=\delta _{n} ( t ) . \end{aligned}$$

By (2.1), (2.15)-(2.17), and Hölder’s inequality, we have

$$\begin{aligned}& \bigl\vert \delta_{n}^{ ( \frac{2}{2-\lambda}-\alpha ) ( 1-\lambda ) }(x)\widetilde{S}_{n,\alpha,\beta}^{\prime} ( f,x ) \bigr\vert \\& \quad \leq Cn\varphi^{ ( \frac{2}{2-\lambda}-\alpha ) ( 1-\lambda ) -2}(x) \biggl( \frac{n+\beta_{2}}{n} \biggr) ^{2n+1} \\& \qquad {}\times\sum_{k=0}^{n}q_{nk}(x) \biggl\vert \frac{k+\alpha_{2}}{n+\beta _{2}}-x\biggr\vert ( n+1 ) \biggl\vert \int_{A_{n}}f \biggl( \frac{nt+\alpha_{1}}{n+\beta_{1}} \biggr) q_{nk}(t)\,dt\biggr\vert \\& \quad \leq Cn\Vert f\Vert _{0}\varphi^{ ( \frac {2}{2-\lambda}-\alpha ) ( 1-\lambda ) -2}(x)\sum _{k=0}^{n}q_{nk}(x)\biggl\vert \frac{k+\alpha_{2}}{n+\beta _{2}}-x\biggr\vert ( n+1 ) \biggl\vert \int_{A_{n}}\delta _{n}^{\alpha ( 1-\lambda ) } ( t ) q_{nk}(t)\,dt\biggr\vert \\& \quad \leq Cn\Vert f\Vert _{0}\varphi^{ ( \frac {2}{2-\lambda}-\alpha ) ( 1-\lambda ) -2}(x)\sum _{k=0}^{n}q_{nk}(x)\biggl\vert \frac{k+\alpha_{2}}{n+\beta _{2}}-x\biggr\vert \biggl( ( n+1 ) \int_{A_{n}}\delta_{n}^{2} ( t ) q_{nk}(t)\,dt \biggr) ^{\alpha ( 1-\lambda ) /2} \\& \qquad {}\times \biggl( ( n+1 ) \int_{A_{n}}q_{nk}(t)\,dt \biggr) ^{1-\alpha ( 1-\lambda ) /2} \\& \quad \leq Cn\Vert f\Vert _{0}\varphi^{ ( \frac {2}{2-\lambda}-\alpha ) ( 1-\lambda ) -2}(x)\sum _{k=0}^{n}q_{nk}(x)\biggl\vert \frac{k+\alpha_{2}}{n+\beta _{2}}-x\biggr\vert \biggl( ( n+1 ) \int_{A_{n}}\delta_{n}^{2} ( t ) q_{nk}(t)\,dt \biggr) ^{\alpha ( 1-\lambda ) /2}. \end{aligned}$$

By (2.9), (2.15) (2.5), and Hölder’s inequality again, we have

2.18 $$\begin{aligned}& \bigl\vert \delta_{n}^{ ( \frac{2}{2-\lambda}-\alpha ) ( 1-\lambda ) }(x)\widetilde{S}_{n,\alpha,\beta}^{\prime} ( f,x ) \bigr\vert \\& \quad \leq Cn\Vert f\Vert _{0}\varphi^{ ( \frac {2}{2-\lambda}-\alpha ) ( 1-\lambda ) -2}(x) \Biggl( \sum_{k=0}^{n}q_{nk}(x)\biggl\vert \frac{k+\alpha_{2}}{n+\beta_{2}}-x\biggr\vert ^{\frac{1}{1-\alpha ( 1-\lambda ) /2}} \Biggr) ^{1-\alpha ( 1-\lambda ) /2} \\& \qquad {}\times \Biggl( \sum_{k=0}^{n}q_{nk}(x) ( n+1 ) \int _{A_{n}}\delta _{n}^{2} ( t ) q_{nk}(t)\,dt \Biggr) ^{\alpha ( 1-\lambda ) /2} \\& \quad \leq Cn^{1/2}\Vert f\Vert _{0} \varphi^{\frac{2 ( 1-\lambda ) }{2-\lambda}-1}(x)\leq Cn^{1/ ( 2-\lambda ) } \Vert f\Vert _{0}. \end{aligned}$$

*Case* 2. $x\in B_{n}^{c}= [ \frac{\alpha_{2}}{n+\beta_{2}},\frac {\alpha _{2}+1}{n+\beta_{2}} ) \cup ( \frac{n+\alpha_{2}-1}{n+\beta _{2}},\frac{n+\alpha_{2}}{n+\beta_{2}} ] $. In this case, we have

2.19 $$ \delta_{n} ( x ) \sim\frac{1}{\sqrt{n}},\quad x\in B_{n}^{c}. $$

Noting that

$$ q_{nk}^{\prime} ( x ) =n \bigl( q_{n-1,k-1} ( x ) -q_{n-1,k} ( x ) \bigr) $$

with $q_{n-1,-1} ( x ) =q_{n-1,n} ( x ) =0$, we get

$$ \widetilde{S}_{n,\alpha,\beta}^{\prime} ( f,x ) =n\sum _{k=0}^{n-1}q_{n-1,k}(x) (x) ( n+1 ) \int_{A_{n}}f \biggl( \frac{nt+\alpha_{1}}{n+\beta_{1}} \biggr) \bigl( q_{n,k+1}(t)-q_{n,k}(t) \bigr) \,dt. $$

Then, by using (2.17) and Hölder’s inequality twice,

2.20 $$\begin{aligned}& \bigl\vert \delta_{n}^{ ( \frac{2}{2-\lambda}-\alpha ) ( 1-\lambda ) }(x)\widetilde{S}_{n,\alpha,\beta}^{\prime} ( f,x ) \bigr\vert \\& \quad \leq Cn\delta_{n}^{ ( \frac{2}{2-\lambda}-\alpha ) ( 1-\lambda ) }(x)\Vert f\Vert _{0}\Biggl\vert \sum_{k=0}^{n-1}q_{n-1,k}(x) ( n+1 ) \int_{A_{n}}\delta _{n}^{\alpha ( 1-\lambda ) } ( t ) \bigl( q_{n,k+1}(t)+q_{n,k}(t) \bigr) \,dt\Biggr\vert \\& \quad \leq Cn\delta_{n}^{ ( \frac{2}{2-\lambda}-\alpha ) ( 1-\lambda ) }(x)\Vert f\Vert _{0}\sum_{k=0}^{n-1}q_{n-1,k}(x) \biggl( ( n+1 ) \int _{A_{n}}\delta _{n}^{2}(t) \bigl( q_{n,k+1}(t)+q_{n,k}(t) \bigr) \,dt \biggr) ^{\frac {\alpha ( 1-\lambda ) }{2}} \\& \quad \leq Cn\delta_{n}^{ ( \frac{2}{2-\lambda}-\alpha ) ( 1-\lambda ) }(x)\Vert f\Vert _{0} \Biggl( \sum_{k=0}^{n-1}q_{n-1,k}(x) ( n+1 ) \int_{A_{n}}\delta _{n}^{2}(t) \bigl( q_{n,k+1}(t)+q_{n,k}(t) \bigr) \,dt \Biggr) ^{\frac {\alpha ( 1-\lambda ) }{2}} \\& \quad \leq Cn\delta_{n}^{ ( \frac{2}{2-\lambda}-\alpha ) ( 1-\lambda ) }(x)\Vert f\Vert _{0}\delta_{n}^{\alpha ( 1-\lambda ) } \\& \quad \leq Cn^{\frac{1}{2-\lambda}} \Vert f\Vert _{0}, \end{aligned}$$

where in the fourth inequality, we used the following fact, which can be deduced exactly in the same way as (2.10):

$$ \sum_{k=0}^{n-1}q_{n-1,k}(x) ( n+1 ) \int_{A_{n}}\delta _{n}^{2}(t)q_{nk^{\ast}}(t) \,dt\leq C\delta_{n}^{2}(x). $$

We obtain (2.13) by combining (2.18) and (2.20).

Now, we begin to prove (2.14). If $( \frac{2}{2-\lambda }-\alpha ) ( \lambda-1 ) <0$, by (2.12) and using Hölder’s inequality twice, we get

$$\begin{aligned}& \bigl\vert \delta_{n}^{ ( \frac{2}{2-\lambda}-\alpha ) ( 1-\lambda ) }(x)\widetilde{S}_{n,\alpha,\beta}^{\prime} ( f,x ) \bigr\vert \\ & \quad \leq C\Vert f\Vert _{1}\Biggl\vert \delta_{n}^{ ( \frac {2}{2-\lambda}-\alpha ) ( 1-\lambda ) }(x)n \sum_{k=0}^{n-1}q_{n-1,k} ( x ) \int_{A_{n}}q_{n+1,k+1}(t)\delta_{n}^{ ( \frac{2}{2-\lambda }-\alpha ) ( \lambda-1 ) }(t) \,dt\Biggr\vert \\ & \quad \leq C\Vert f\Vert _{1}\delta_{n}^{ ( \frac {2}{2-\lambda}-\alpha ) ( 1-\lambda ) }(x) \sum_{k=0}^{n-1}q_{n-1,k} ( x ) \biggl( n \int_{A_{n}}q_{n+1,k+1}(t)\delta_{n}^{-2}(t) \,dt \biggr) ^{\frac{1}{2} ( \frac{2}{2-\lambda}-\alpha ) ( 1-\lambda ) } \\ & \qquad {}\times \biggl( n \int_{A_{n}}q_{n+1,k+1}(t)\,dt \biggr) ^{1-\frac {1}{2} ( \frac{2}{2-\lambda}-\alpha ) ( 1-\lambda ) } \\ & \quad \leq C\Vert f\Vert _{1}\delta_{n}^{ ( \frac {2}{2-\lambda}-\alpha ) ( 1-\lambda ) }(x) \sum_{k=0}^{n-1}q_{n-1,k} ( x ) \biggl( n \int_{A_{n}}q_{n+1,k+1}(t)\delta_{n}^{-2}(t) \,dt \biggr) ^{\frac{1}{2} ( \frac{2}{2-\lambda}-\alpha ) ( 1-\lambda ) } \\ & \qquad (\text{by (2.1)}) \\ & \quad \leq C\Vert f\Vert _{1}\delta_{n}^{ ( \frac {2}{2-\lambda}-\alpha ) ( 1-\lambda ) }(x) \Biggl( \sum_{k=0}^{n-1}q_{n-1,k} ( x ) n \int _{A_{n}}q_{n+1,k+1}(t)\delta _{n}^{-2}(t) \,dt \Biggr) ^{\frac{1}{2} ( \frac{2}{2-\lambda}-\alpha ) ( 1-\lambda ) } \\ & \quad \leq C\Vert f\Vert _{1}, \end{aligned}$$

where, in the last inequality, (2.10) is applied.

If $( \frac{2}{2-\lambda}-\alpha ) ( \lambda-1 ) >0$, by using (2.9) instead of (2.10), we also can deduce that

$$ \bigl\vert \delta_{n}^{ ( \frac{2}{2-\lambda}-\alpha ) ( 1-\lambda ) }(x)\widetilde{S}_{n,\alpha,\beta}^{\prime} ( f,x ) \bigr\vert \leq C\Vert f\Vert _{1}. $$

 □

### Lemma 7

*If* $0\leq\lambda\leq1$, $0<\alpha<2$, *then*

2.21 $$\begin{aligned}& \bigl\Vert \widetilde{S}_{n,\alpha,\beta} ( f ) \bigr\Vert _{2}\leq Cn\Vert f\Vert _{0}, \quad f\in C_{\alpha ,\lambda}, \end{aligned}$$

2.22 $$\begin{aligned}& \bigl\Vert \widetilde{S}_{n,\alpha,\beta} ( f ) \bigr\Vert _{2}\leq C\Vert f\Vert _{2}, \quad f\in C_{\alpha,\lambda }^{2}. \end{aligned}$$

### Proof

It can be proved in a way similar to Lemma 6. □

### Lemma 8

*For* $0< t<\frac{1}{8}$, $\frac{t}{2}\leq x\leq1-\frac{t}{2}$, $x\in [ 0,1 ] $, $\beta<2$, *we have*

2.23 $$ \int_{-t/2}^{t/2}\delta_{n}^{-\beta} ( x+u ) \, du\leq C(\beta )t\delta_{n}^{-\beta}(x). $$

### Lemma 9

*For* $0< t<\frac{1}{4}$, $t\leq x\leq1-t$, $x\in [ 0,1 ] $, $0\leq \beta\leq2$, *we have*

2.24 $$ \int_{-t/2}^{t/2} \int_{-t/2}^{t/2}\delta_{n}^{-\beta} ( x+u+v ) \,du\,dv\leq Ct^{2}\delta_{n}^{-\beta}(x). $$

It has been shown in [9] that Lemma 8 and Lemma 9 are valid when $\delta_{n} ( t ) $ is replaced by $\delta_{n}^{\ast} ( t ) $, which combining with (2.8) proves Lemma 8 and Lemma 9.

## Proofs of theorems

### Proof of Theorem 1

Define the auxiliary operators $\mathbf{S}_{n,\alpha,\beta} ( f,x ) $ as follows:

3.1 $$ \mathbf{S}_{n,\alpha,\beta} ( f,x ) =\widetilde {S}_{n,\alpha ,\beta} ( f,x ) +L_{n,\alpha,\beta} ( f,x ) , $$

where

$$ L_{n,\alpha,\beta} ( f,x ) =f(x)-f \bigl( \widetilde {S}_{n,\alpha ,\beta} ( t,x ) \bigr) . $$

By (2.3) and (2.4), we have

3.2 $$\begin{aligned}& \bigl\vert \widetilde{S}_{n,\alpha,\beta} ( t,x ) -x \bigr\vert \leq \frac{C}{n}, \end{aligned}$$

3.3 $$\begin{aligned}& \mathbf{S}_{n,\alpha,\beta} ( 1,x ) =1,\qquad \mathbf{S}_{n,\alpha,\beta} ( t-x,x ) =0, \end{aligned}$$

and

3.4 $$ \Vert \mathbf{S}_{n,\alpha,\beta} \Vert \leq3. $$

It follows from (3.2) that

3.5 $$ \bigl\vert L_{n,\alpha,\beta} ( f,x ) \bigr\vert \leq\omega \bigl( f,\bigl\vert \widetilde{S}_{n,\alpha,\beta} ( t,x ) -x\bigr\vert \bigr) \leq C\omega \biggl( f,\frac{1}{n} \biggr) . $$

From (1.7) and (1.8), for any fixed *x*, *λ*, and *n*, we may choose a $g_{n,x,\lambda} ( t ) \in\overline{D}_{\lambda}^{2} $ such that

3.6 $$\begin{aligned}& \Vert f-g\Vert \leq C\omega_{\varphi^{\lambda}}^{2} \bigl( f,n^{-1/2}\delta_{n}^{1-\lambda}(x) \bigr) , \end{aligned}$$

3.7 $$\begin{aligned}& \bigl( n^{-1/2}\delta_{n}^{1-\lambda}(x) \bigr) ^{2}\bigl\Vert \varphi ^{2\lambda}g^{\prime\prime}\bigr\Vert \leq C\omega_{\varphi ^{\lambda }}^{2} \bigl( f,n^{-1/2} \delta_{n}^{1-\lambda}(x) \bigr) , \end{aligned}$$

3.8 $$\begin{aligned}& \bigl( n^{-1/2}\delta_{n}^{1-\lambda}(x) \bigr) ^{4/ ( 2-\lambda ) }\bigl\Vert g^{\prime\prime}\bigr\Vert \leq C \omega_{\varphi ^{\lambda}}^{2} \bigl( f,n^{-1/2} \delta_{n}^{1-\lambda}(x) \bigr) . \end{aligned}$$

By (3.4) and (3.6), we have

3.9 $$\begin{aligned} \bigl\vert \mathbf{S}_{n,\alpha,\beta} ( f,x ) -f(x)\bigr\vert \leq&\bigl\vert \mathbf{S}_{n,\alpha,\beta } \bigl( ( f-g ) ,x \bigr) \bigr\vert +\bigl\vert f(x)-g(x)\bigr\vert +\bigl\vert \mathbf{S}_{n,\alpha,\beta} ( g,x ) -g(x)\bigr\vert \\ \leq&4\Vert f-g\Vert +\bigl\vert \mathbf{S}_{n,\alpha ,\beta} ( g,x ) -g(x) \bigr\vert \\ \leq&C\omega_{\varphi^{\lambda}}^{2} \bigl( f,n^{-1/2}\delta _{n}^{1-\lambda}(x) \bigr) +\bigl\vert \mathbf{S}_{n,\alpha,\beta } ( g,x ) -g(x)\bigr\vert . \end{aligned}$$

Noting that $\varphi^{2\lambda}(x)$ and $\delta_{n}^{2\lambda}(x)$ are concave functions on $[ 0,1 ] $, for any $t,x\in [ 0,1 ] $, and *u* between *x* and *t*, say $u=\theta x+ ( 1-\theta ) t$, $0\leq\theta\leq1$, we have

3.10 $$\begin{aligned}& \frac{\vert t-u\vert }{\varphi^{2\lambda}(u)}=\frac{\theta \vert t-x\vert }{\varphi^{2\lambda}(\theta x+ ( 1-\theta ) t)}\leq\frac{\theta \vert t-x\vert }{\theta\varphi ^{2\lambda}(x)+ ( 1-\theta ) \varphi^{2\lambda}(t)}\leq \frac{\vert t-x\vert }{\varphi^{2\lambda}(x)}, \end{aligned}$$

3.11 $$\begin{aligned}& \frac{\vert t-u\vert }{\delta_{n}^{2\lambda}(u)}\leq\frac{ \vert t-x\vert }{\delta_{n}^{2\lambda}(x)}. \end{aligned}$$

By using Taylor’s expansion

$$ g(t)=g(x)+g^{\prime}(x) ( t-x ) + \int_{x}^{t} ( t-u ) g^{\prime\prime}(u)\,du, $$

(3.3), and (3.11),

$$\begin{aligned} \bigl\vert \mathbf{S}_{n,\alpha,\beta} ( g,x ) -g(x)\bigr\vert =&\biggl\vert \mathbf{S}_{n,\alpha,\beta} \biggl( \int_{x}^{t} ( t-u ) g^{\prime\prime}(u)\,du,x \biggr) \biggr\vert \\ \leq&\biggl\vert \widetilde{S}_{n,\alpha,\beta} \biggl( \int _{x}^{t} ( t-u ) g^{\prime\prime}(u)\,du,x \biggr) \biggr\vert \\ &{}+\biggl\vert \int_{x}^{\widetilde{S}_{n,\alpha,\beta} ( t,x ) } \bigl( \widetilde{S}_{n,\alpha,\beta} ( t,x ) -u \bigr) g^{\prime \prime }(u)\,du\biggr\vert . \end{aligned}$$

When $x\in B_{n}$, by (2.15), (3.10), (3.2), and (2.2), we have

3.12 $$\begin{aligned} \bigl\vert \mathbf{S}_{n,\alpha,\beta} ( g,x ) -g(x)\bigr\vert \leq&C\bigl\Vert \varphi^{2\lambda}g^{\prime \prime }\bigr\Vert \widetilde{S}_{n,\alpha,\beta} \biggl( \frac{ ( t-x ) ^{2}}{\varphi^{2\lambda}(x)},x \biggr) +\varphi^{-2\lambda}(x) \bigl\Vert \varphi^{2\lambda}g^{\prime\prime}\bigr\Vert \bigl( \widetilde{S}_{n,\alpha,\beta} ( t,x ) -x \bigr) ^{2} \\ \leq&Cn^{-1}\delta_{n}^{2-2\lambda}(x)\bigl\Vert \varphi^{2\lambda }g^{\prime\prime}\bigr\Vert \\ \leq&C\omega_{\varphi^{\lambda}}^{2} \bigl( f,n^{-1/2}\delta _{n}^{1-\lambda}(x) \bigr) , \end{aligned}$$

where in the last inequality, (3.7) is applied.

When $x\in B_{n}^{c}$, by (2.19), (3.10), (3.2), and (2.2), we have

3.13 $$\begin{aligned} \bigl\vert \mathbf{S}_{n,\alpha,\beta} ( g,x ) -g(x)\bigr\vert \leq&C\bigl\Vert \delta_{n}^{2\lambda}g^{\prime \prime }\bigr\Vert \widetilde{S}_{n,\alpha,\beta} \biggl( \frac{ ( t-x ) ^{2}}{\delta_{n}^{2\lambda}(x)},x \biggr) + \delta_{n}^{-2\lambda }(x)\bigl\Vert \delta_{n}^{2\lambda}g^{\prime\prime} \bigr\Vert \bigl( \widetilde{S}_{n,\alpha,\beta} ( t,x ) -x \bigr) ^{2} \\ \leq&Cn^{-1}\delta_{n}^{2-2\lambda}(x) \biggl( \bigl\Vert \varphi ^{2\lambda}g^{\prime\prime}\bigr\Vert +\frac{1}{n^{\lambda}} \bigl\Vert g^{\prime\prime}\bigr\Vert \biggr) \\ \leq&Cn^{-1}\delta_{n}^{2-2\lambda}(x)\bigl\Vert \varphi^{2\lambda }g^{\prime\prime}\bigr\Vert +C \bigl( n^{-1/2} \delta_{n}^{1-\lambda }(x) \bigr) ^{4/ ( 2-\lambda ) }\bigl\Vert g^{\prime\prime }\bigr\Vert \\ \leq&C\omega_{\varphi^{\lambda}}^{2} \bigl( f,n^{-1/2}\delta _{n}^{1-\lambda}(x) \bigr) , \end{aligned}$$

where in the last inequality, we used (3.7) and (3.8).

We complete the proof of Theorem 1 by combining (3.1), (3.5), (3.9), (3.12), and (3.13).

### Proof of Theorem 2

With Lemma 6-Lemma 9, the proof of Theorem 2 can be found exactly in the same way as that of [9]. We omit the details here.

## References

[1] Gadjiev AD, Ghorbanalizaeh AM (2010). Approximation properties of a new type Bernstein-Stancu polynomials of one and two variables. Appl. Math. Comput..

[2] Stancu DD (1968). Approximation of functions by a new class of linear polynomial operators. Rev. Roum. Math. Pures Appl..

[3] Wang ML, Yu DS, Zhou P (2014). On the approximation by operators of Bernstein-Stancu types. Appl. Math. Comput..

[4] İçöz G (2012). A Kantorovich variant of a new type Bernstein-Stancu polynomials. Appl. Math. Comput..

[5] Taşdelen F, Başcanbaz-Tunca G, Erençin A (2012). On a new type Bernstein-Stancu operators. Fasc. Math..

[6] Jung HS, Deo N, Dhamija M (2014). Pointwise approximation by Bernstein type operators in mobile interval. Appl. Math. Comput..

[7] Acar T, Aral A, Gupta V (2015). On approximation properties of a new type Bernstein-Durrmeyer operators. Math. Slovaca.

[8] Ditzian Z, Totik V (1987). Moduli of Smoothness.

[9] Guo S, Liu L, Liu X (2001). The pointwise estimate for modified Bernstein operators. Studia Sci. Math. Hung..

